# Interim Estimates of Vaccine Effectiveness of Pfizer-BioNTech and Moderna COVID-19 Vaccines Among Health Care Personnel — 33 U.S. Sites, January–March 2021

**DOI:** 10.15585/mmwr.mm7020e2

**Published:** 2021-05-21

**Authors:** Tamara Pilishvili, Katherine E. Fleming-Dutra, Jennifer L. Farrar, Ryan Gierke, Nicholas M. Mohr, David A. Talan, Anusha Krishnadasan, Karisa K. Harland, Howard A. Smithline, Peter C. Hou, Lilly C. Lee, Stephen C. Lim, Gregory J. Moran, Elizabeth Krebs, Mark Steele, David G. Beiser, Brett Faine, John P. Haran, Utsav Nandi, Walter A. Schrading, Brian Chinnock, Daniel J. Henning, Frank LoVecchio, Joelle Nadle, Devra Barter, Monica Brackney, Amber Britton, Kaytlynn Marceaux-Galli, Sarah Lim, Erin C. Phipps, Ghinwa Dumyati, Rebecca Pierce, Tiffanie M. Markus, Deverick J. Anderson, Amanda K. Debes, Michael Lin, Jeanmarie Mayer, Hilary M. Babcock, Nasia Safdar, Marc Fischer, Rosalyn Singleton, Nora Chea, Shelley S. Magill, Jennifer Verani, Stephanie Schrag, Vaccine Effectiveness Among Healthcare Personnel Study Team, Vaccine Effectiveness Among Healthcare Personnel Study Team, Anna Yousaf, Yunmi Chung, Jennifer Onukwube, Wei Xing, Bradley Clinansmith, Lisandra Uribe, Kye E. Poronsky, Dean M. Hashimoto, Monica Bahamon, Michelle St. Romain, Efrat Kean, Amy Stubbs, Sara Roy, Gregory Volturo, James Galbraith, James C. Crosby, Megan R. Fuentes, Mary Mulrow, Jane Lee, Helen Johnston, AmberJean Hansen, Scott K. Fridkin, Lucy E. Wilson, Sara Lovett, Melissa Christian, Christopher Myers, Valerie L.S. Ocampo, Keipp Talbot, Jessica Seidelman, Aaron M. Milstone, Mary Hayden, Matthew Samore, Jennie H. Kwon, Daniel Shirley, Denise Dillard, Jennifer Dobson

**Affiliations:** ^1^CDC COVID-19 Response Team; ^2^University of Iowa, Iowa City, Iowa; ^3^Olive View–University of California Los Angeles Medical Center, Los Angeles, California; ^4^Baystate Medical Center, Springfield, Massachusetts; ^5^Brigham and Women’s Hospital, Boston, Massachusetts; ^6^Jackson Memorial Hospital, Miami, Florida; ^7^Louisiana State University, New Orleans, Louisiana; ^8^Thomas Jefferson University Hospital, Philadelphia, Pennsylvania; ^9^Truman Medical Center, Kansas City, Missouri; ^10^University of Chicago, Chicago, Illinois; ^11^University of Massachusetts, Worcester, Massachusetts; ^12^University of Mississippi, Jackson, Mississippi; ^13^University of Alabama at Birmingham, Birmingham, Alabama; ^14^University of California San Francisco, Fresno, California; ^15^University of Washington, Seattle, Washington; ^16^Valleywise Health Medical Center, Phoenix, Arizona; ^17^California Emerging Infections Program, Oakland, California; ^18^Colorado Department of Public Health and Environment, Denver, Colorado; ^19^Connecticut Emerging Infections Program, Yale School of Public Health, New Haven, Connecticut; ^20^Georgia Emerging Infections Program, Atlanta, Georgia; ^21^Emory University School of Medicine, Atlanta, Georgia; ^22^Maryland Department of Health, Baltimore, Maryland; ^23^Minnesota Emerging Infections Program, Minnesota Department of Health, St. Paul, Minnesota; ^24^University of New Mexico, Albuquerque, New Mexico; ^25^New Mexico Emerging Infections Program, Santa Fe, New Mexico; ^26^University of Rochester Medical Center/New York State—Rochester Emerging Infections Program, Rochester, New York; ^27^Public Health Division Oregon Health Authority, Portland, Oregon; ^28^Vanderbilt University Medical Center, Nashville, Tennessee; ^29^Duke Center for Antimicrobial Stewardship and Infection Prevention, Duke University School of Medicine, Durham, North Carolina; ^30^Johns Hopkins School of Public Health, Baltimore, Maryland; ^31^Department of Medicine, Rush University Medical Center, Chicago, Illinois; ^32^University of Utah, VA Salt Lake City Health Care System, Salt Lake City, Utah; ^33^Washington University School of Medicine, St. Louis, Missouri; ^34^University of Wisconsin—Madison, Madison, Wisconsin; ^35^William S. Middleton Memorial Veterans Hospital, Madison, Wisconsin; ^36^Alaska Native Tribal Health Consortium, Anchorage, Alaska.; CDC COVID-19 Response Team; CDC COVID-19 Response Team; CDC COVID-19 Response Team; CDC COVID-19 Response Team; University of Iowa, Iowa City, Iowa; Olive View—University of California Los Angeles Medical Center, Los Angeles, California; Baystate Medical Center, Springfield, Massachusetts; Brigham and Women’s Hospital, Boston, Massachusetts; Jackson Memorial Hospital, Miami, Florida; Louisiana State University, New Orleans, Louisiana; Thomas Jefferson University Hospital, Philadelphia, Pennsylvania; Truman Medical Center, Kansas City, Missouri; University of Chicago, Chicago, Illinois; University of Massachusetts, Worcester, Massachusetts; University of Mississippi, Jackson, Mississippi; University of Alabama at Birmingham, Birmingham, Alabama; University of Washington, Seattle, Washington; Valleywise Health Medical Center, Phoenix, Arizona; California Emerging Infections Program, Oakland, California; Colorado Department of Public Health and Environment, Denver, Colorado; Connecticut Emerging Infections Program, Yale School of Public Health, New Haven, Connecticut;; Georgia Emerging Infections Program, Atlanta, Georgia, and Emory University School of Medicine, Atlanta, Georgia; Department of Emergency Health Services, University of Maryland, Baltimore County, Baltimore Maryland; Minnesota Emerging Infections Program, Minnesota Department of Health, St. Paul, Minnesota; University of New Mexico, Albuquerque, New Mexico, and New Mexico Emerging Infections Program, Santa Fe, New Mexico; University of Rochester Medical Center/New York State—Rochester Emerging Infections Program,; Rochester, New York; Public Health Division, Oregon Health Authority, Portland, Oregon; Vanderbilt University Medical Center, Nashville, Tennessee; Duke Center for Antimicrobial Stewardship and Infection Prevention, Duke University School of Medicine, Durham, North Carolina; Johns Hopkins School of Public Health, Baltimore, Maryland; Department of Medicine, Rush University, Medical Center, Chicago, Illinois; University of Utah, VA Salt Lake City Health Care System, Salt Lake City, Utah; Washington University School of Medicine, St. Louis, Missouri; University of Wisconsin—Madison, Madison, Wisconsin; Southcentral Foundation, Anchorage, Alaska; Yukon-Kuskokwim Health Corporation, Bethel, Alaska

Throughout the COVID-19 pandemic, health care personnel (HCP) have been at high risk for exposure to SARS-CoV-2, the virus that causes COVID-19, through patient interactions and community exposure (*1*). The Advisory Committee on Immunization Practices recommended prioritization of HCP for COVID-19 vaccination to maintain provision of critical services and reduce spread of infection in health care settings (*2*). Early distribution of two mRNA COVID-19 vaccines (Pfizer-BioNTech and Moderna) to HCP allowed assessment of the effectiveness of these vaccines in a real-world setting. A test-negative case-control study is underway to evaluate mRNA COVID-19 vaccine effectiveness (VE) against symptomatic illness among HCP at 33 U.S. sites across 25 U.S. states. Interim analyses indicated that the VE of a single dose (measured 14 days after the first dose through 6 days after the second dose) was 82% (95% confidence interval [CI] = 74%–87%), adjusted for age, race/ethnicity, and underlying medical conditions. The adjusted VE of 2 doses (measured ≥7 days after the second dose) was 94% (95% CI = 87%–97%). VE of partial (1-dose) and complete (2-dose) vaccination in this population is comparable to that reported from clinical trials and recent observational studies, supporting the effectiveness of mRNA COVID-19 vaccines against symptomatic disease in adults, with strong 2-dose protection.

A test-negative design case-control study of mRNA COVID-19 VE is underway, with HCP being enrolled at 33 sites across 25 U.S. states; the planned interim analysis presented in this report includes data collected during January–March 2021.* A majority (75%) of enrolled HCP worked at acute care hospitals (including emergency departments), 25% worked in outpatient or specialty clinics, and <1% worked in long-term care facilities and urgent care clinics. HCP with the potential for exposure to SARS-CoV-2 through direct patient contact or for indirect exposure (e.g., through infectious materials) were eligible for enrollment.^†^ Case-patients and control participants (controls) were identified through routine employee testing performed based on site-specific occupational health practices. HCP with a positive SARS-CoV-2 polymerase chain reaction (PCR) or antigen-based test result and at least one COVID-19–like illness symptom^§^ were enrolled as case-patients, and HCP with a negative SARS-CoV-2 PCR test result, regardless of symptoms, were eligible for enrollment as controls. Controls were frequency matched to case-patients (aiming for a ratio of three controls per case-patient) by site and week of test. HCP who reported having received a positive SARS-CoV-2 PCR or antigen-based test result >60 days earlier (i.e., with a previous SARS-CoV-2 infection) were excluded. Information on demographics, COVID-19–like illness symptoms within 14 days before or after the testing date, and presence of underlying conditions and risk factors for severe COVID-19^¶^ were collected through HCP interviews or self-completed surveys. Medical records were reviewed to collect data on SARS-CoV-2 test dates, type, and results and on medical care sought for COVID-19–like illness. Vaccination records, including dates and type of COVID-19 vaccine received, were obtained from occupational health or other verified sources (e.g., vaccine card, state registry, or medical record).

HCP were defined as unvaccinated if they had not received any COVID-19 vaccine doses or had received their first dose after the test date. The interval of 0–13 days from receipt of the first dose was defined as the time before first dose vaccine effect. The effectiveness of a single dose was measured during the interval from 14 days after the first dose through 6 days after the second dose. Because of the potential for vaccine-related reactions to influence HCP testing behaviors, sensitivity analyses of single-dose VE were conducted 1) excluding participants tested within 0–2 days of receiving the second dose and 2) measuring VE before receiving the second dose. Effectiveness of 2 doses was measured ≥7 days after the receipt of the second dose, consistent with the Pfizer-BioNTech clinical trial procedure (*3*). Sensitivity analyses measuring 2-dose effectiveness ≥14 days after the second dose were conducted, consistent with the Moderna clinical trial procedure (*4*). Conditional logistic regression was used to estimate matched odds ratios (mORs) adjusted for age, race/ethnicity, and presence of underlying conditions. VE was estimated as 100% × (1–mOR) for 1 or 2 doses, compared with no doses. Because of the small sample size, analyses could not be stratified by COVID-19 vaccine type. All statistical analyses were conducted using SAS (version 9.4; SAS Institute). This activity was reviewed by CDC and was conducted consistent with applicable federal law and CDC policy.**

As of March 18, 2021, 623 case-patients and 1,220 controls had been enrolled. The median ages of case-patients and controls were 38 years (range = 19–69 years) and 37 years (range = 19–76 years), respectively (Table 1). The majority of HCP (60% of case-patients and 64% of controls) worked in occupational categories with substantial anticipated direct patient contact and were aged 19–49 years (75% and 76%, respectively), female (84% and 82%, respectively), and non-Hispanic White (64% and 70%, respectively). Underlying conditions associated with increased risk for severe COVID-19 were reported by 77% of case-patients and 75% of controls. Case-patients were significantly more likely than controls to have fever (40% versus 23%, p<0.001), cough (56% versus 22%, p<0.001), or shortness of breath (26% versus 7%, p<0.001); 5% of case-patients and 14% of controls reported only mild symptoms (sore throat, headache, runny nose, or congestion; p<0.001); 17% of controls reported no symptoms. Only 12 (2%) case-patients and 10 (1%) controls had severe illness requiring hospitalization, and no deaths occurred in either group.

**TABLE 1:** Characteristics of health care personnel case-patients and controls — 33 U.S. sites, January–March 2021

Characteristic	No. (%)	
	Case-patients* (N = 623)	Controls* (N = 1,220)
**Age group, yrs**		
**Median (range)**	**38 (19–69)**	**37 (19–76)**
19–49	470 (75)	931 (76)
50–64	144 (23)	257 (21)
≥65	7 (1)	24 (2)
Missing	2(<1)	8 (<1)
**Sex**		
Male	99 (16)	223 (18)
Female	521 (84)	996 (82)
Other	3 (<1)	1 (<1)
**Race/Ethnicity**		
White, non-Hispanic	401 (64)	853 (70)
Black, non-Hispanic	64 (10)	64 (5)
Hispanic/Latino	81 (13)	124 (10)
Other^†^	77 (13)	179 (15)
**Anticipated level of HCP patient contact based on occupational category**		
Substantial^§^	375 (60)	785 (64)
Moderate^¶^	60 (10)	120 (10)
Minimal**	147 (24)	221 (18)
Undefined^††^	41 (7)	94 (8)
**Presence of one or more underlying conditions or risk factors associated with increased risk for severe COVID-19** ^§§^	480 (77)	920 (75)
Obesity (BMI >30 kg/m^2^ or listed in medical record)	217 (35)	395 (32)
Overweight (BMI 25–29 kg/m^2^ or listed in medical record)	186 (30)	355 (29)
Asthma	98 (16)	211 (17)
Hypertension	92 (15)	159 (13)
Diabetes mellitus^¶¶^	28 (4)	57 (5)
Immunocompromising condition***	25 (4)	46 (4)
Heart disease	15 (2)	61 (5)
Cerebrovascular disease	2 (<1)	4 (<1)
Neurologic condition	2 (<1)	7 (<1)
Chronic kidney disease	1 (<1)	5 (<1)
Chronic obstructive pulmonary disease	1 (<1)	6 (<1)
Other chronic lung disease	6 (<1)	16 (1)
Chronic liver disease	2 (<1)	6 (<1)
Current or former smoking^†††^	130 (21)	255 (21)
Pregnancy (proportion among female HCP)	13 (3)	40 (4)
**Reported symptoms of illness**		
Fever (measured temperature ≥100.4°F [38.0°C] or subjective)^§§§^	249 (40)	281 (23)
Cough (dry or productive)^§§§^	348 (56)	267 (22)
Shortness of breath^§§§^	161 (26)	80 (7)
Chills^§§§^	275 (44)	324 (27)
Muscle pain^§§§^	289 (46)	342 (28)
Altered sense of smell or taste^§§§^	351 (56)	45 (4)
Sore throat^§§§^	215 (35)	344 (28)
Diarrhea^§§§^	154 (25)	173 (14)
Nausea or vomiting^§§§^	132 (21)	186 (15)
Other symptoms^¶¶¶^	560 (90)	796 (65)
**Hospitalized**	12 (2)	10 (1)
**COVID-19 vaccine status**		
Unvaccinated	340 (55)	302 (25)
Received ≥1 dose before test date, by vaccine type	283 (45)	918 (75)
Pfizer-BioNTech	214 (76)	712 (78)
Moderna	68 (24)	200 (22)
Mixed product****	0	1 (0.4)
Missing product information	1 (0.4)	5 (0.5)

**Abbreviations:** HCP = health care personnel; PCR = polymerase chain reaction.

* Case-patients: HCP who received positive SARS-CoV-2 PCR or antigen-based test results and had one or more symptoms of COVID-19–like illness; controls: HCP who received negative SARS-CoV-2 PCR test results.

^†^ Includes Asian or Pacific Islander (44 case-patients, 109 controls), American Indian or Alaska Native (23 case-patients, 35 controls), multiple races (5 case-patients, 19 controls), and missing race (5 case-patients, 16 controls).

^§^ Substantial patient contact occupational categories: health care providers (physicians, residents, fellows, attending physicians, nurse practitioners, and physician assistants), nurses (registered nurses, other nursing providers including intensive care unit nurses, nurse managers, and midwives), direct patient assistants (licensed practical nurses, certified nursing assistants, patient care technicians and assistants, medical assistants, COVID-19 testers, phlebotomists, home health care providers, emergency medical services providers, and paramedics), and medical therapists (physical therapists; physical therapy assistants; rehabilitation providers; rehabilitation aides; occupational therapists; speech and language pathologists; respiratory therapists; radiology technicians; dental health care providers, including dentists or dental hygienists; and surgical, medical, or emergency technicians).

^¶^ Moderate patient contact occupational categories: behavioral/social services providers (behavioral health providers [excluding physician psychiatrists], chaplains, social workers and assistants, care coordinators, interpreters, patient registration personnel, health educators, genetic counselors, ambulance dispatchers, dieticians, and research staff members), and environmental services providers (facilities staff members, food services workers, transport workers, patient transport workers, and drivers).

** Minimal patient contact occupational categories: administrative or ward clerks, symptom checkers, telehealth trainers, clinical support staff members, equipment and sterile processing technicians, medical equipment sales personnel, laboratory personnel, and pharmacists.

^††^ Undefined patient contact occupational categories: others who could not be classified into any of the preceding categories and those with missing information.

^§§^ Conditions associated with definite or potential increased risk for severe COVID-19 illness as defined by CDC. https://www.cdc.gov/coronavirus/2019-ncov/need-extra-precautions/people-with-medical-conditions.html?CDC_AA_refVal=https%3A%2F%2Fwww.cdc.gov%2Fcoronavirus%2F2019-ncov%2Fneed-extra-precautions%2Fgroups-at-higher-risk.html

^¶¶^ Among HCP who reported diabetes mellitus, no case-patients and two controls (<1% of all controls) reported type 1 diabetes, eight case-patients (1% of all case-patients) and nine controls (<1% of all controls) reported type 2 diabetes, and 20 case-patients (3%) and 46 controls (4%) did not specify a diabetes type.

*** Immunocompromising conditions include immunosuppression medication (e.g., corticosteroids, chemotherapy, or other immunosuppressive medications), solid organ transplant, hematopoietic stem cell transplant, HIV, thalassemia, or active cancer (current cancer or in treatment or received diagnosis within last 12 months).

^†††^ Smoking includes cigarettes, tobacco, e-cigarettes/vaping, or marijuana use.

^§§§^ Statistically significant difference between case-patients and controls; chi-square test, p-value<0.001.

^¶¶¶^ Other symptoms include chest pain or tightness, abdominal pain, loss of appetite, red or bruised toes or feet, headache, runny nose, or congestion.

**** One person’s first dose was Moderna vaccine and second dose was Pfizer-BioNTech vaccine.

Ten percent of case-patients and 20% of controls had received 1 dose of COVID-19 vaccine ≥14 days before the test date, and 3% of case-patients and 15% of controls had received 2 doses ≥7 days before the test date (Table 2). Among vaccinated persons, 76% of case-patients and 78% of controls received the Pfizer-BioNTech vaccine; the remainder received the Moderna vaccine. The adjusted single-dose VE was 82% (95% CI = 74%–87%) and was similar for both 1-dose sensitivity analyses (before dose 2: VE = 74%, 95% CI = 62%–82%; excluding days 0–2 after dose 2: VE = 78%, 95% CI = 68%–84%). The adjusted 2-dose VE was 94% (95% CI = 87%–97%); effectiveness ≥14 days after the second dose was similar (VE = 90%, 95% CI = 77%–96%).

**TABLE 2:** COVID-19 vaccine effectiveness among health care personnel case-patients and controls, by number of COVID-19 vaccine doses received before SARS-CoV-2 test date — 33 U.S. sites, January–March 2021

Interval from dose to test date	No. (%)		Vaccine effectiveness^†^ % (95% CI)	
	Case-patients* (N = 623)	Controls* (N = 1,220)	Unadjusted	Adjusted^§^
**Dose 1**				
≥14 days	64 (10)	241 (20)	82.2 (75.1–87.3)	81.7 (74.3–86.9)
**Dose 2**				
≤2 days	5 (<1)	109 (9)		
3–6 days	16 (3)	85 (7)		
≥7 days	19 (3)	184 (15)	93.4 (86.4–96.8)	93.5 (86.5–96.9)

**Abbreviations:** CI = confidence interval; HCP = health care personnel; mOR = matched odds ratio; OR = odds ratio; PCR = polymerase chain reaction; VE = vaccine effectiveness.

* Case-patients: HCP who received positive SARS-CoV-2 PCR or antigen-based test results and had one or more symptoms of COVID-19–like illness; controls: HCP who received negative SARS-CoV-2 PCR test results.

^†^ VE (Pfizer-BioNTech and Moderna) was estimated using a conditional logistic regression model accounting for matching by site of enrollment and week of test date.

^§^ OR used in conditional logistic regression model to calculate VE was adjusted for age, race, and presence of underlying conditions: VE = 100% × (1−mOR).

## Discussion

This multisite test-negative design case-control study found that authorized mRNA COVID-19 vaccines (Pfizer-BioNTech and Moderna) are highly effective against symptomatic COVID-19 among HCP. Effectiveness of a complete 2-dose regimen of these vaccines was estimated to be 94%, consistent with findings from two clinical trials (*3*,*4*). Although the case definition applied in this study was broader than that used in both clinical trials (*3*,*4*), 93% and 88% of cases included in this study met the respective Pfizer-BioNTech and Moderna trial case definitions. The results are also consistent with findings from an observational study among the general adult population from Israel (*5*), two cohort studies among HCP from the United Kingdom,^††^ and recently reported interim results from a U.S. cohort evaluation among HCP and frontline workers (*6*).

Effectiveness of a single dose, estimated to be 82% in this report, has also been demonstrated in phase III trials and recent observational studies. The estimated effectiveness found in this report is higher than estimates of single-dose effectiveness found in the Pfizer-BioNTech clinical trial (efficacy 52%; 95% CI = 30%–68%) (*3*) and an observational study from Israel (*5*). In the Israeli study, the Pfizer-BioNTech VE against symptomatic illness among the general adult population was 57% (95% CI = 50%–63%) and 66% (95% CI = 57%–73%) measured during 14–20 and 21–27 days, respectively, after the first dose (*5*). These differences might be related to the younger age of the HCP population in this study (<2% of participants aged ≥65 years) compared with the age of the Israeli study population (13% aged ≥70 years). In two cohort studies among HCP, the single-dose effectiveness of the Pfizer-BioNTech vaccine was consistent with the estimates in this report, with 72% effectiveness (95% CI = 58%–86%) 21 days after the first dose in a U.K. study (*7*) and 80% effectiveness (95% CI = 59%–90%) ≥14 days after the first dose in a U.S. cohort study (*6*). Because the single-dose effectiveness estimates in this and other studies were based on a short follow-up, the duration of this level of protection from a single dose is unknown.

The findings in this report are subject to at least four limitations. First, testing for SARS-CoV-2 infection among HCP was based on occupational health practices at each facility, and no changes in routine testing practices were reported after vaccine introduction. If vaccinated HCP were less likely to obtain testing than unvaccinated HCP, the VE might have been underestimated. Alternatively, if postvaccination reactions increased the likelihood that vaccinated HCP would seek testing, the VE might have been overestimated. However, the sensitivity analysis excluding the interval of 0–2 days after receipt of dose 2, the interval during which most postvaccination reactions would be expected to occur, did not significantly change effectiveness estimates. Second, because of the limited sample size, effectiveness by vaccine product, presence of underlying medical conditions, and disease severity could not be estimated. In addition, because of limited statistical power, effectiveness estimates could not be adjusted for other potential confounders, such as use of personal protective equipment, occupational categories, or workplace or community exposures. Third, the VE estimates might not be generalizable to the U.S. adult population because racial/ethnic minority groups disproportionately affected by COVID-19 and who may have had higher exposure risks in the community were underrepresented in this population, and the overall HCP population was younger than the general U.S. adult population. However, the study’s geographic coverage was broad, representing the population of U.S. HCP, and vaccination data were obtained from multiple sources. Finally, although HCP with a known past acute SARS-CoV-2 infection were excluded, those whose previous infection was unknown could not be excluded. Data collection for this study is ongoing and will allow effectiveness to be evaluated by vaccine type and among HCP subgroups.

These interim results demonstrate that complete vaccination with authorized mRNA COVID-19 vaccines is highly effective in preventing symptomatic COVID-19 among HCP, supporting the results of phase III trials and additional accruing evidence in recent observational studies. Real-world VE data are critical to guiding evolving COVID-19 vaccine policy. In addition to adherence to recommended infection control and prevention practices, a critical component of controlling the U.S. COVID-19 pandemic and protecting HCP is ensuring high coverage with safe and effective COVID-19 vaccines.

**Summary**

**What is already known about this topic?**

Health care personnel (HCP) are at high risk for COVID-19. The early distribution of two mRNA COVID-19 vaccines (Pfizer-BioNTech and Moderna) to HCP provided an opportunity to examine vaccine effectiveness in a real-world setting.

**What is added by this report?**

The first U.S. multisite test-negative design vaccine effectiveness study among HCP found a single dose of Pfizer-BioNTech or Moderna COVID-19 vaccines to be 82% effective against symptomatic COVID-19 and 2 doses to be 94% effective.

**What are the implications for public health practice?**

The mRNA vaccines are highly effective at preventing symptomatic COVID-19 among U.S. HCP. High vaccination coverage among HCP and the general population is critical to prevent COVID-19 in the United States.

## Footnotes

*
https://www.cdc.gov/vaccines/covid-19/downloads/hcp-early-phase-protocol-508.pdf

† https://www.cdc.gov/coronavirus/2019-ncov/hcp/guidance-risk-assesment-hcp.html 

§ Health care personnel are considered symptomatic if one or more of the following signs and symptoms are present 14 days before or after the test date: fever (documented ≥100.4°F [38.0°C] or subjective), chills, cough (dry or productive), shortness of breath, chest pain or tightness, fatigue or malaise, sore throat, headache, runny nose, congestion, muscle aches, nausea or vomiting, diarrhea, abdominal pain, altered sense of smell or taste, loss of appetite, or red or bruised toes or feet.

¶ Underlying conditions grouped based on CDC guidelines identifying conditions associated or potentially associated with risk for severe COVID-19 illness. https://www.cdc.gov/coronavirus/2019-ncov/need-extra-precautions/people-with-medical-conditions.html

** This investigation was defined as having met the requirements for public health surveillance as defined in 45 C.F.R. part 46.102(l)(2) 21 C.F.R. part 56; 42 U.S.C. Sect. 241(d); 5 U.S.C. Sect. 552a; 44 U.S.C. Sect. 3501 et seq.

†† https://doi.org/10.1101/2021.03.09.21253218; https://doi.org/10.1101/2021.03.11.21253275
